# Avian Emotions: Comparative Perspectives on Fear and Frustration

**DOI:** 10.3389/fpsyg.2018.02707

**Published:** 2019-01-17

**Authors:** Mauricio R. Papini, Julio C. Penagos-Corzo, Andrés M. Pérez-Acosta

**Affiliations:** ^1^ Texas Christian University, Fort Worth, TX, United States; ^2^ Universidad de las Américas Puebla, Cholula, Mexico; ^3^ Universidad del Rosario, Bogotá, Colombia

**Keywords:** emotion, fear, frustration, birds, aggression, response suppression, conflict, comparative psychology

## Abstract

Emotions are complex reactions that allow individuals to cope with significant positive and negative events. Research on emotion was pioneered by Darwin’s work on emotional expressions in humans and animals. But Darwin was concerned mainly with facial and bodily expressions of significance for humans, citing mainly examples from mammals (e.g., apes, dogs, and cats). In birds, emotional expressions are less evident for a human observer, so a different approach is needed. Understanding avian emotions will provide key evolutionary information on the evolution of related behaviors and brain circuitry. Birds and mammals are thought to have evolved from different groups of Mesozoic reptiles, theropod dinosaurs and therapsids, respectively, and therefore, their common ancestor is likely to be a basal reptile living about 300 million years ago, during the Carboniferous or Permian period. Yet, birds and mammals exhibit extensive convergence in terms of relative brain size, high levels of activity, sleep/wakefulness cycles, endothermy, and social behavior, among others. This article focuses on two basic emotions with negative valence: fear and frustration. Fear is related to the anticipation of dangerous or threatening stimuli (e.g., predators or aggressive conspecifics). Frustration is related to unexpected reward omissions or devaluations (e.g., loss of food or sexual resources). These results have implications for an understanding of the conditions that promote fear and frustration and for the evolution of supporting brain circuitry.

## Introduction

In one of his epistles to Lucius, the Roman philosopher Seneca wrote that “No animal, when it enters upon life, is free from the fear of death” (Seneca, Epistle CXXI, 19, trans., Seneca, 1925). This conclusion is consistent with the general observation that most animals seem to have evolved withdrawal, escape, and avoidance behaviors from threatening situations, and seek and approach situations that promote their well-being and fulfill their reproductive potential. For example, in the mollusk *Tritonia diomedea*, tactual stimulation by a starfish (a natural predator) triggers a series of dorsoventral contractions that allow the animal to escape to a distant location (Wyeth and Willows, 2006). In some cases, however, animals seem to be willing to die, especially in defense of their colony. For example, when the colony is attacked by a predator, postreproductive females of the aphid *Quadrartus yoshinomiyai* attach themselves to the predator and secrete a sticky substance that glues the intruder and also themselves to the plant. This kills the predator, but also results in the aphid’s death (Uematsu et al., 2010). Are these behaviors accompanied by a subjective emotional experience?

Educated people may find it difficult to believe that organisms considered simple, such as a mollusk and an aphid, have the neural sophistication sufficient to produce emotional responses. But what about animals with more complex nervous systems, such as mammals and birds? Comparative psychologists have struggled with the problem of emotion because, as many other important concepts, they cannot be directly observed or measured. Darwin (1871/1965) took a different approach by emphasizing emotional *expressions*, that is, the facial and bodily movements that accompany situations that seemed akin to those that would generate emotional responses in humans. Darwin cited examples mainly drawn from mammals (e.g., apes, dogs, and cats). For example, “animals which live in society often call to each other when separated, and evidently feel much joy at meeting; as we see with a horse, on the return of his companion, for whom he has been neighing” (Darwin, 1871/1965, pp. 84–85). In Darwin’s book, descriptions of emotions in birds are reduced to a few examples of negative valence. Birds “ruffled their feathers when angry or frightened” (p. 97) and are “accustomed, when in danger, either to squat on the ground or to sit motionless on a branch, so as to escape detection” (p. 100). Whereas contemporary researchers generally reject such an anthropomorphic language (but see, Safina, 2015), most would still recognize Darwin’s two descriptions of avian emotion given above as examples of agonistic behavior and fear-induced freezing. Curiously, Darwin provided no examples of avian emotions that could be related to frustration, disappointment, or loss, although the example with reunited horses also given above points to the effects of social separation in a mammal.

In defining these emotions, we continue to have limits similar to those encountered by Darwin in 1871. First, we still cannot study animal emotions directly because we have no access to an organism’s subjective experience. Therefore, we must depend on animal behavior and physiology—emotional expression. Second, we have to use standards developed by those who study emotional behavior in mammals and apply them to other taxonomic groups. Third, the human emotional repertoire seems too broad to start a discussion of animal emotion. It seems more effective to center our attention in a few emotions that have clear protective function. In this review, we focus on two such emotions: fear and frustration.

Animals are said to be under the influence of fear if they “avoid when they can, flee if avoidance has not been successful, and defend themselves, usually aggressively, when flight is impossible or difficult” (Gray and McNaughton, 2000, p. 38). The avoidance of threat may involve response suppression (usually called “freezing”), which, as noted by Darwin, helps the organism to avoid detection. Frustration tends to evoke similar behaviors, but in response to the unexpected failure of rewards to occur. Thus, frustration “is a temporary state that results when a response is nonreinforced (or nonrewarded in more neutral language in the appetitive case) in the presence of a reward expectancy” (Amsel, 1992, p. 1). Amsel characterizes the behavioral consequences of frustration in terms of four descriptive concepts: invigoration, suppression, persistence, and regression. When either fear or frustration is combined with approach responses triggered by the same location or stimulus, they also contribute to anxiety. For example, fear of a location previously paired with pain (electric shock) does not induce anxiety, unless the organism must approach that location for some other reason (e.g., food or social proximity). Traditionally, this is characterized as an approach-avoidance conflict (Miller, 1944). Anxiety is dependent upon the conflict induced by an ambivalent goal.

This article reviews evidence from studies with birds based on situations designed to study fear and frustration, and the ensuing states of anxiety and conflict. But first, we ask what is a bird and what do we know about their evolutionary history?

## What is a Bird?

Although birds and mammals have evolved from different reptilian lineages (theropod dinosaurs and therapsids, respectively; Benton, 2014), there are many phenotypic similarities that originated independently by convergent evolution (Kemp, 1988). Some characters common to birds and mammals that are not found in extant reptiles include relatively large brain size, similar general activity levels, cycles of sleep and wakefulness, endothermy, and complex patterns of reproductive behavior, among others. Convergent evolution in learning has been explicitly postulated for movement mimicry among psittacine birds and primates (Moore, 1992). Is it possible that evolutionary convergence extends to the emotions of fear and frustration?

The fossil record suggests that by the late Jurassic period, about 140–150 million years ago, there were several lineages of theropod dinosaurs with feathers. A specimen from China, *Anchiornis huxleyi*, dated 155 million years old, was a small theropod dinosaur covered with feathers, although not likely capable of flight (Hu et al., 2009). There is increasing evidence that feathers first evolved in dinosaurs for functions unrelated to flight, such as thermoregulation or display. Consistent with their use in communication displays, a study of melanosomes, molecules responsible for feather color in living birds, suggested that *Anchiornis* was brightly colored with gray, reddish-brown, white, and black feathers (Li et al., 2010). Although not considered a bird, *Anchiornis* and a few other fossil specimens, are classified in the same group with *Archaeopteryx lithographica*, a transition form with reptilian and avian characters, and all of them as the immediate ancestors of birds (Benton, 2014).

*Archaeopteryx* was not like any modern bird. It had teeth, a bony and long tail, and claws, all reptilian characters, and although the feathers had the asymmetric morphology typical of modern birds with flight capacity, whether it could take off from the ground up remains controversial (Voeten et al., 2018). Modern birds appeared in the fossil record during the Cenozoic era (Benton, 2014), but mitochondrial DNA studies suggest an origin during the late Cretaceous period, around 100 million years ago (Brown et al., 2008). *Vegavis iaai* is a fossil bird from the Late Cretaceous of Antarctica, dated 66–68 million years ago and considered to be related to modern birds (Clarke et al., 2005). This evidence suggests that the ancestors of modern birds survived the mass extinction event of the Cretaceous-Paleogene boundary, dated to about 65 million years ago, and then underwent an adaptive radiation during the early portion of the Cenozoic era that parallels that of the mammals.

Both birds and mammals show evidence of encephalization, that is, relatively large brain size, compared to reptiles (Striedter, 2005; Northcutt, 2011). *Archaeopteryx* also appears to have had a relatively large brain (Balanoff et al., 2013), although perhaps not as large as that of modern birds (Alonso et al., 2004). Encephalization in birds and mammals has proceeded independently from reptilian ancestors that, as far as we know, have a relative brain size that, on average, was about 10 times smaller (Northcutt, 2011). Avian encephalization may be related to a number of characters, such as endothermy, flight, cognition, and sociality. Daily energy demands that could sustain some of these functions may have triggered a positive feedback loop with brain size favoring the evolution of encephalization. It is clear that the organization of the telencephalon of birds and mammals has proceeded independently to a point that it is difficult to recognize homologies at a macroanatomical level. Equally clearly, however, there are substantial telencephalic homologies that can be appreciated at the level of afferent-efferent connections, neurochemical systems, and gene-expression profiles (Montiel and Aboitiz, 2018).

Cognitive skills and social behavior have been connected to brain evolution and high levels of anatomical change in birds (Wyles et al., 1983; Iwaniuk and Wylie, 2017). For example, tool use is correlated with increased size of the avian (nidopallium) and primate (neocortex) brain (Mehlhorn et al., 2010; Lefebvre, 2013). A similar connection between brain and emotion is only beginning to be made (Panksepp, 2017). In part, the relative paucity of results is due to a large extent on the fact that behavioral data on emotion in birds are not clear. One goal of this review is to bring this issue to the foreground.

## Avian Fear

Fear and related emotional states are induced in response to threat, whether actual or potential. The following review is organized into four areas of research involving threatening stimuli: response suppression, active escape/avoidance behavior, aggressive behavior, and conflict.

### Response Suppression

Birds exhibit freezing and corticosterone release in fear-inducing situations. In one study (de Haas et al., 2012), freezing behavior (lack of motion and vocalizations) was assessed in an open field in young chicks and the effects were measured at an adult age. Freezing levels positively correlated with adult corticosterone levels. Moreover, corticosterone increased in birds subjected to a test involving restriction of movement. Interestingly, the presence of a fearful bird increased corticosterone levels in other individuals within the group. Additional research on response suppression in birds relates to tonic immobility (TI). TI involves the suppression of the righting response, reduced vocalizations, and intermittent eye closure (Pusch et al., 2018). In a TI paradigm, the experimenter holds the hen by hand upside down for 15 s and then releases the animal. TI occurs if no movement is detected for 10 s (Hrabcakova et al., 2012; Pusch et al., 2018). According to Gallup (1973), TI is a reliable index of fear in birds. He found that a conditioned stimulus for shock is more effective than a shock to increase the duration of TI. A recent review concluded that TI remains a valid indicator of affective states in the face of threats (Fureix and Meagher, 2015). Quail strains selected for high and low TI show differences in fear responses, with the high TI strain showing more freezing and less exploration than the low TI strain (Jones et al., 1991). These differences were attenuated by exposure to environmental enrichment (colored geometric patterns painted on cards and pinned to the walls and a variety of colored objects).

The posterior arcopallium in birds (corresponding to portions of the mammalian amygdala; Jarvis et al., 2005; Hanics et al., 2017) seems to play an important role in the control of fear responses, as it does in mammals (Ressler and Maren, 2019). In Japanese quail, for example, lesions of the posterior arcopallium increase behavior indicative of fear, whereas lesions of the anterior arcopallium reduce fear responses (Saint-Dizier et al., 2009). Fear responses were tested in four tasks: TI, open-field test, hole-in-the-wall test, and novel-object test. In addition, there is evidence of lateralization of function in the arcopallium. In chickens, unilateral lesions of the right medial arcopallium have a greater effect on fear responses than equivalent lesions in the left hemisphere (Phillips and Youngren, 1986).

Neophobia is the rejection of novel objects. In birds, neophobia is usually measured in terms of the latency to approach, sit on, and finally take a food item from a novel feeder. Black-capped chickadees (*Poecile atricapillus*) and European starlings (*Sturnus vulgaris*) exhibited longer latency to approach and consume food from a novel feeder than from a familiar feeder (Apfelbeck and Raess, 2008; Roth et al., 2010). Aversive conditioning also potentiates neophobia. In an experiment with chicks (Franchina and Dryer, 1989), novel visual (red water) or taste (vinager) simuli were paired with an injection of lithium chloride, a toxin that induces sickness, or a saline injection. In subsequent neophobia tests, animals were tested with either green water (visual novelty) or saline water (taste novelty). There was evidence of neophobia in both tests, but conditioning with the vinager led to more neophobia than conditioning with red water. Bobwhite quail (*Colinus virginianus*) can be easily conditioned to suppress consumption of food with a novel color after pairings with a toxin (Wilcoxon et al., 1971).

Passive avoidance research with birds has attracted more attention. In one procedure, beads of a specific color are coated with a substance that has an aversive taste to chickens (methyl anthranilate). In later tests, young chicks are given successive tests with beads of the same or different color and the amount of pecking is recorded. Chicks peck more at the novel color than at the color paired with the aversive substance, but the order in which the colors are presented and the time between successive tests determine the outcome of the test (Crowe and Hale, 2002).

Several aspects of passive avoidance learning in chicks are controlled by different parts of the telencephalon. For example, extensive lesions of the dorsal, lateral, and anterior hyperpallium (formerly hyperstriatum) impaired the retention and relearning of passive avoidance. Interestingly, whereas lesions of the dorsal hyperpallium disrupted acquisition of passive avoidance, anterior lesions caused no measurable deficits in passive avoidance (Benowitz, 1972). More recent research has focused on neurochemical and cellular effects of passive avoidance learning. The administration of dopamine agonists in one-day-old chicks alters memory consolidation in this passive avoidance task (Hale and Crowe, 2002). In the same species, this passive avoidance procedure resulted in an increase in cellular proliferation in the mesopallium ventrale (formerly hyperstriatum ventrale) and in the olfactory tubercle (Dermon et al., 2002). However, passive avoidance has also been reported to produce a decrease in hippocampal synaptic density in chickens (Nikolakopoulou et al., 2006). This may imply that passive avoidance is a stressful event leading to dendritic atrophy and thus a reduced spine density. Others reported the opposite result, namely, an increase in spine density in the right hemisphere of the hippocampus of male chicks trained in passive avoidance (Unal et al., 2002). The source of these differences is unclear. What is clear is that passive avoidance training in chickens gives rise to an aversive memory that has measurable neural correlates similar to those observed in mammals. For example, the density of hippocampal synaptic spines is also modified by Pavlovian fear conditioning in mice (Abate et al., 2018).

Circadian factors are also important in passive avoidance. Chickens that received a single pairing between a red bead and methyl anthranilate at 16:00 h had poor discrimination (i.e., they generalized responding) in red-versus-blue tests administered 24 h later. By contrast, chicks trained at 08:00 h and 12:00 h discriminated red from blue beads more accurately (Radford et al., 1981). Experiments with mammals show similar sensitivity to circadian factors (Barbachano et al., 2017). Caging conditions also affect passive avoidance learning. Using electric shocks as reinforcer, chickens caged in groups exhibited better learning than animals housed in individual cages (Brown, 1976). Group caging is known to also facilitate aversive learning in mammals (Penagos-Corzo et al., 2015).

### Active Escape/Avoidance Behavior

Active escape/avoidance behavior has been studied in a variety of situations. This section covers research on alarm signals, the flight initiation distance (FID), and active escape/avoidance learning.

The social learning of predator avoidance can be conceptualized in conditioning terms. The predator is the conditioned stimulus and the social alarm of a conspecific is the unconditioned stimulus. Accordingly, stimuli associated to social alarm are tagged as threatening. However, at least in some birds, learned social avoidance is temporarily more flexible than in laboratory paradigms of conditioning in which a stimulus predicts important biological events (Griffin, 2004, 2008). A natural predator not always generates automatic avoidance. For instance, there is evidence from several bird species that some animals are attracted to the predator and the experience derived from these encounters is used by conspecifics. Observing a predator model with a dead gull (*Larus argentatus*) increased the avoidance distance of gulls in a later encounter with the model (Kruuk, 1976). In many species, the encounter with a predator releases specific behaviors that conspecifics perceive and later use to tag the stimulus as threatening. Social alarm in birds is based on calls and also on the noise of the wings at take-off flight (Hingee and Magrath, 2009). Social alarm in birds may be conspecific or heterospecific (Dawson Pell et al., 2018). In the case of Japanese tits (*Parus minor*), birds that have learned to respond to alarm calls become more responsive to objects resembling the predator—a sign of a search image (Suzuki, 2018). It is also worth noting that birds show the acquisition of passive avoidance through observation. For example, chickens can learn about an aversive object observing the responses of another chicken (Johnston et al., 1998). Thus, chicks that observed through a wire mesh the aversive reaction of another chick when pecking at a bead coated with methyl anthranilate later avoided a similar bead. Avoidance learning by observation has also been reported in natural environments in great tits (Landová et al., 2017; Thorogood et al., 2018).

The FID has also been used in natural environments to assess escape/avoidance behavior (Møller, 2010). Increased fear in birds, as in mammals, is associated with adrenal cortex activation (Davis et al., 2008; Tilgar et al., 2010). Neuroendocrine responses involve hypothalamic–pituitary–adrenal (HPA) axis activation and corticosterone secretion (Cockrem, 2007). Even if there is evidence supporting the relationship of both FID and corticosterone with stress and fear, the relationship between these two variables is not always direct. For example, in a comparison between urban and rural birds, despite finding differences in the FID between these two populations, a relationship with corticosterone was not found (Rebolo-Ifrán et al., 2015).

In the traditional instrumental paradigm of active avoidance learning, a signal precedes an aversive stimulus, unless the animal makes a specific response during the signal, in which case, the signal is terminated and the aversive stimulus is prevented. There are two versions of active avoidance learning, one in which the animal moves to a different compartment when the warning signal is presented. This procedure is called two-way avoidance because either compartment is both safe and dangerous depending on the trial. In the second procedure, called one-way avoidance, the animal also moves to another compartment, but each compartment is always either safe or dangerous. Macphail (1968) provided evidence of both types of avoidance learning in pigeons, albeit with only a few animals and with sequential training starting with one-way avoidance and ending with two-way avoidance training. Pigeons also exhibited efficient acquisition of a treadle-pressing response to avoid shock in an unsignaled Sidman avoidance procedure (Smith and Keller, 1970). This procedure is analogous to active avoidance, except that no explicit signal is presented (shocks and response feedback provide signals). Instead, shocks occur at regular intervals and a response postpones the next shock for a period longer than the shock-shock interval. Using a visual + auditory compound and a treadle response, Foree and LoLordo (1975) reported that pigeons learned to respond to prevent punishment for pecking at a key for food. Moreover, post-training tests determined that the treadle response was controlled predominantly by the visual element of the compound. Interestingly, when pigeons pressed a treadle just to avoid shock or just to obtain food, then behavior was controlled predominantly by the auditory or visual component, respectively (Foree and LoLordo, 1973). Lesions of the arcopallium (previously archistriatum) impaired both active two-way avoidance and Sidman avoidance learning in pigeons (Dafters, 1975), just as they also impaired passive avoidance (see above). Two-way active avoidance performance is actually enhanced by section of the olfactory nerves, even though the warning signal was visual (Hutton et al., 1974). Opioid receptors in the olfactory tubercle (previously associated to the lobus paraolfactorius) have also been detected in chicks trained in the passive avoidance paradigm. Post-training infusions of mu and delta opioid-receptor antagonists in the olfactory tubercle impaired retention of the passive avoidance task, but kappa and opioid receptor-like receptor antagonists had no effect (Freeman and Young, 2000).

### Aggressive Behavior

Experiments with several mammalian species have shown that aggressive behavior can be elicited by painful stimuli, such as electric shocks (Ulrich, 1966). Pigeons, which readily peck at other pigeons (or at stuffed pigeons or mirror images) during periods of nonreinforcement (see below), do not seem to respond with aggressive behavior toward a stuffed conspecific when receiving shocks (Rashotte et al., 1974). Such discrepancy can be understood in terms of selective pressures related to foraging and predator avoidance. Pigeons compete with conspecifics for access to food, but they rarely engage in intraspecific aggressive behaviors. Consistent with this characterization, pigeons show aggressive responses to conspecifics in the context of foraging (e.g., appetitive extinction), but not in the context of defensive behavior (e.g., shock-induced pain).

Aggressive behavior occurs in chickens reared in animal production facilities. For a variety of reasons, chickens show an aggressive behavior described as feather pecking that results in bleeding and even cannibalism (Fossum et al., 2009). Feather pecking has been associated to fear responses in the target animal. Although birds show agitation after the initial attacks, this behavior is followed by periods of freezing. Physiological changes suggest that feather pecking induces pain (Gentle and Hunter, 1991). In a line of birds selected for low mortality from feather pecking, Kops et al. (2013) reported lower levels of noradrenaline activity in the arcopallium relative to a control line and, consistent with this finding, lower levels of fear in the manual restriction test (in this test, the animal is tilted on its right side while its legs are gently pulled; response movements are recorded).

### Conflict

Conflict situations usually involve a convergence of two contingencies of opposite hedonic value (Miller, 1944). This can be implemented in a Skinner box situation, where pigeons trained in a fixed-ratio or variable-ratio schedule for food reinforcement (which generates high rates of key pecking) are concurrently exposed to punishment with shock contingent on some key-pecking responses. A number of studies show that while punishment leads to behavioral suppression, there is also a tendency for recovery of response levels within the session (Dardano, 1970; Powell, 1970).

There is extensive psychopharmacological research on the effects of a variety of drugs on punished responding in birds. Chlordiazepoxide (CDP), an anxiolytic drug that binds to the benzodiazepine site in the GABA_A_ receptor and facilitates the influx of chloride ions induced by GABA (Meyer and Quenzer, 2018), increases punished responding in rats, monkeys, and pigeons (Barrett and Gleeson, 1991). McMillan (1973) reported that CDP and diazepam (also a benzodiazepine anxiolytic) increase response rate under punished responding relative to matched unpunished behavior. Punished responding is also increased by bretazenil (a partial agonist of the benzodiazepine site of GABA receptors), which does not affect matched nonpunished responding, and the effect was eliminated by the benzodiazepine antagonist flumazenil (Witkin et al., 1997).

A variety of serotoninergic drugs have been used in the treatment of anxiety and depression in humans, but have little or no effects in established animal models of either disorders with nonhuman mammals. Yet, pigeons respond to these drugs much like human patients (Barrett et al., 1994). One example is buspirone (a serotonin-1A-receptor agonist with anxiolytic effects). Buspirone does not affect punished responding in rats (Howard and Pollard, 1990) or squirrel monkeys (Wettstein, 1988), and it also fails to eliminate consummatory negative contrast in rats (Flaherty et al., 1990). However, buspirone eliminates the suppressive effects of punishment in pigeons (Barrett and Witkin, 1991). The mechanism of action is benzodiazepine-independent (Barrett et al., 1986), but serotonin-dependent (Witkin et al., 1987; Gleeson et al., 1989).

The evidence reviewed in this section is consistent with the hypothesis that at least some avian species experience emotional states akin to mammalian fear. The behavioral, neurobiological, and psychopharmacological results reviewed above suggest interesting parallels between mammals and birds in emotional behavior.

## Avian Frustration

Following the lead from experiments with rodents, this review focuses on a variety of procedures involving some form of surprising reward omission (SRO; Papini and Dudley, 1997). Such events are labeled as “surprising” because they occur in the presence of signals or in situations previously associated with reward presentation. SROs can lead to a variety of behavioral and physiological effects in rodents, including response invigoration, response elicitation, escape, response suppression, and persistence. In this section, we ask whether similar behavioral effects have been observed in analogous experiments with birds. This evidence will provide a base to test the hypothesis that birds experience emotions akin to frustration when exposed to SROs. The following sections are organized along six areas of research: response invigoration, response suppression, escape/avoidance behavior, persistence, aggressive behavior, and conflict.

### Response Invigoration

Amsel and Roussel (1952) reported that rats ran faster after a surprising nonreward event than after a surprising reward event in a double-runway apparatus. Their interpretation is that a SRO induces an emotional state (called primary frustration), which energizes behavior by increasing motivation to engage the dominant response. A major problem of interpretation has been to distinguish between response invigoration after the SRO (frustration) versus response suppression after the reward presentation (transient demotivation). Subsequent research has shown that both processes, response invigoration and suppression, actually occur in situations involving food omission (Stout et al., 2003). The emotional interpretation is consistent also with the elimination of response invigoration during early appetitive extinction trials in rats deprived of adrenal glands and, therefore, of reduced circulating glucocorticoids (Thomas and Papini, 2001). Adrenalectomized rats learn and extinguish lever-pressing behavior, but lack the response invigoration that usually occurs when food is initially withheld in extinction.

Response invigoration (increased key-pecking behavior) after occasional food omissions has produced inconsistent results in pigeons. In free-operant procedures, Staddon and Innis (1969) reported response invigoration when rewards were omitted in fixed-interval schedules. However, Wilton et al. (1969) reported inconsistent results also using free-operant schedules in pigeons, although the same procedures produced response invigoration in rats. Interestingly, Wilton et al. (1969) observed pacing behavior following reward omission, which they interpreted as indicating an emotional effect of the omission. In an additional phase, Wilton et al. (1969) introduced a limited hold on responding, such that pigeons had to peck the target key within a 0.9-s period to be able to obtain a reward. This limited hold was introduced to reduce the influence of pacing. Under these conditions, the results were again inconclusive. One pigeon showed response invigoration, one showed response suppression, and a third one exhibited no clear effect of reward omissions. However, even if a consistent difference in responding after reward versus after nonreward would have been obtained, the issue of whether it was invigoration driven by frustration or suppression caused by transient demotivation would have remained unresolved.

More conclusive effects of reward omissions were reported in discrete-trial experiments. For example, during fixed-interval performance, pigeons exhibited higher key-pecking rates after reward omission than after reward presentations. However, increasing the intertrial interval from 2 to 12 s eliminated the effect by increasing performance after both outcomes relative to a group receiving continuous reinforcement (Papini and Hollingsworth, 1998). A frustration account would have been supported by a reduction in responding during the long intertrial interval, but, instead, performance was increased, a result pointing to transient demotivation. Thus, pigeons responded less after food presentation, rather than more after food omission. Similar results were obtained in a runway experiment. Pigeons could anticipate rewarded and nonrewarded trials when discriminative stimuli were used, but not when outcomes were unpredictable. However, they ran faster after nonreward than after reward whether the outcomes were expected or unexpected (Stout et al., 2002). Again, this effect seems to have been caused by transient demotivation to respond for food. Furthermore, key pecking was higher immediately after nonreward than after reward, a difference that was eliminated by introducing a delay for responding. However, the difference was eliminated by response recovery after surprising reward, rather than by response decay after surprising nonreward, as a frustrative interpretation anticipated (Stout et al., 2002). Thus, there is no evidence that pigeons (or any other avian species, as far as the authors are aware) exhibit response invigoration after a SRO event that is attributable to primary frustration.

### Response Suppression

As with rats (Logan, 1960; Rashotte and Amsel, 1968), pigeons find it difficult to inhibit responding for food when response spacing is required for reinforcement. For example, pigeons have difficulty adjusting to a schedule of differential reinforcement of low rates in which key pecking needs to occur once every 20 s for reward to be delivered (McMillan and Campbell, 1970). In addition, both d-amphetamine (a drug with multiple synaptic effects, including inhibition of monoamine uptake; Meyer and Quenzer, 2018) and CDP had a small, disrupting effect on key pecking during such schedules. These drug effects are difficult to interpret due to the small number of birds used with each dose (*n* = 2) and to the inconsistency of performance. Perhaps, the most parsimonious explanation for the small disrupting effect of both drugs would appeal to a motor impairment, rather than an emotional component. In general, animals find it difficult to inhibit responding for food, even when a cost (i.e., reward loss) is inflicted upon such impulsive behavior. In pigeons, however, key pecking shows greater impulsivity than treadle pressing (pushing a lever with a foot) in a differential reinforcement of low rates situation (Hemmes, 1975). Whereas pigeons continued operating a treadle with up to a 35-s delay requirement for responding, key pecking was disrupted by a delay requirement as short as 14 s. Nonetheless, examples of response suppression in appetitive situations are especially interesting because the behavior has to overcome the tendency toward impulsive responding.

Response suppression is expected if an animal learns to anticipate, based on prior experience, either the forthcoming omission of a reward or the frustrative response such an omission caused, both having negative emotional connotations. Such learning occurs, for example, during appetitive extinction when approach responses decrease to a minimum, as has been shown since Pavlov (1927) in a variety of situations. Emotional responses can modulate the speed of the response decrement occurring during appetitive extinction. So, rats trained with large versus small rewards and shifted to extinction exhibit differential rates of response decrement—faster after large reward than after small reward acquisition (Wagner, 1961; Papini et al., 2001). One explanation of this phenomenon, called the magnitude of reinforcement extinction effect (MREE), is that the omission of a large reward conditions a stronger anticipation of frustration than the omission of a small reward and, therefore, a stronger avoidance of the goal that accelerates extinction (Amsel, 1992).

Pigeons trained to traverse a runway or to peck at a key in a spaced-trial situation (one trial per day) showed evidence of a reversed MREE, that is, faster extinction after training with a small reward than with a large reward (Papini, 1997; Papini and Thomas, 1997; Thomas and Papini, 2003). Interestingly, pigeons trained with different cues signaling the large and small magnitudes in a successive, spaced-trial discrimination procedure (a spaced-trial version of a simultaneous contrast paradigm) also extinguished more slowly after acquisition with the large-reward cue, than with the small-reward cue (Papini, 1997). A similar reversed MREE was reported in Japanese quail (*Coturnix japonica*) trained in a sexual conditioning paradigm (Baquero et al., 2009). In this case, groups of quail received access to either eight females or one receptive female in the goal box of a runway. When shifted to extinction (no females present at the goal), large reward led to slower extinction than small reward.

In the MREE paradigm, groups trained with different reward magnitudes (large vs. small) are shifted to the same magnitude in extinction (no reward). This is formally similar to the successive negative contrast (SNC) paradigm in which the shift is to a lower, but nonzero, magnitude. In this situation, a reward downshift from a large to a small magnitude has a transient suppressive effect on approach behavior relative to an unshifted control always trained with the small reward (Flaherty, 1996). SNC is related to a variety of indicators suggesting that the effect is accompanied by an aversive emotional reaction—primary and anticipatory frustration, depending on the situation (Papini et al., 2015).

Pigeons trained to peck at an illuminated key for 15 food pellets showed shorter response latencies than pigeons trained to peck for one food pellet, a fact indicating that the magnitudes were discriminable. However, a 15-to-1 pellet downshift caused only a gradual adjustment of response latencies without any signs of SNC (Papini, 1997). This result is consistent with the reversed MREE effects described above; in both cases, pigeons exhibited difficulty to suppress behavior following SROs. An experiment with chickens using a wet cereal reward (more preferred) versus the same cereal mixed with orange oil (less preferred) in a runway also produced evidence of a reversed SNC effect (Davis et al., 2015). These results may not be general across avian species since evidence of SNC was reported in starlings (*S. vulgaris*). In one experiment (Freidin et al., 2009), two groups of starlings received either mealworms (a preferred food) or turkey crumbs during preshift trials. Mealworm animals consumed more food than turkey-crumb animals, thus showing that the rewards affected consummatory behavior. Then, the mealworm animals were downshifted to turkey crumbs, whereas the unshifted controls continued to receive turkey crumbs. Now, downshifted animals consumed less than unshifted controls, thus showing evidence for SNC. In addition, probing the target (i.e., inserting its beak into the bowl that used to contain mealworms in preshift trials) produced no evidence of SNC. Such probing behavior is likely to have been instrumentally reinforced by access to mealworms, in which case, a failure to observe SNC in probing is potentially significant.

Instrumental behavior provided evidence of reversed MREE and SNC in three avian species (pigeons, quail, and starlings), but a regular SNC effect was observed in terms of consummatory behavior in starlings. In rats, consummatory SNC effects tend to be more robust than instrumental SNC effects. For example, several experiments in which rats were trained with sucrose solutions of different concentrations in the goal box of a runway have produced evidence of consummatory, but not instrumental, SNC in the same animals (Flaherty and Caprio, 1976; Sastre et al., 2005). Similarly, the same rats that produced no evidence of SNC in single-option training yielded evidence of SNC in free-choice trials in which they could respond to two levers previously paired with large and small rewards (Conrad and Papini, 2018). In free-choice trials, rats preferred the lever associated to the large reward during preshift, but switched preference to the lever associated with the unshifted reward during postshift trials. As a result, it seems plausible that implementing a SNC manipulation using either consummatory or free-choice procedures would yield evidence of frustration in birds.

### Escape Behavior

The previous section provided scanty evidence for response suppression in situations involving reward downshifts. Suppression of approach behavior is akin to goal avoidance. One can also ask whether birds demonstrate evidence of escaping from a stimulus situation in which they are either exposed to surprising nonreward (escape from frustration effect; Norris et al., 2009) or to a signal for nonreward (escape from the S− effect; Terrace, 1971). To our knowledge, there are no demonstrations of the escape from frustration effect in birds, but there is evidence for the escape from the S− effect in pigeons.

The aversive properties of surprising nonreinforcement can be assessed directly. For example, pigeons learn a key-pecking response when its only consequence is to terminate the stimulus predicting no reward (S−), in an S+/S− successive discrimination (Rilling et al., 1969; Terrace, 1971; Gonzalez and Champlin, 1974). For example, Rilling et al. (1969) trained pigeons in a multiple schedule in which one component delivered rewards at a high rate, whereas the other component was either extinction or had a lower reward rate. Pigeons had the opportunity to peck at a second key at any time during the session and turn off all the lights in the conditioning box. Under these conditions, pigeons pecked more frequently at this second key when they were either in extinction or reinforced at a lower rate. Since the escape key did not affect the rate of reinforcement, these results cannot be interpreted in terms of reducing the delay to the next reward. Terrace (1971) also showed that the removal of the escape contingency results in the extinction of this response. Interestingly, Terrace (1971) reported that escape from the S− does not develop following errorless discrimination training in which the pigeon has never experienced nonreinforcement for responding to the S− and, therefore, should not have experienced frustration. These results were interpreted uniformly as suggesting that the S− has aversive emotional properties.

Pigeons also learn to postpone a time-out period from reinforcement much as they learn to postpone an electric shock. Such responses may be considered to be examples of active avoidance, as different from the response suppression examples discussed in the previous section. Pigeons were trained under a concurrent schedule in which pecking at one key produced rewards on a variable interval schedule while pecking at a second key postponed a time-out period during which all lights were turned off. Consistent with previous studies with rats and chimpanzees, DeFulio and Hackenberg (2007) reported that pigeons acquired responding to the second key that postponed a time-out period, but reduced such responses when they did not affect the occurrence of the time-out period. In addition, they showed that stimuli paired with effective postponement of the time out induced higher response rates than stimuli that were less effective in postponing the time out. The authors view this procedure in terms of the aversive properties of the time-out period. To the extent that a time out postpones food delivery, it has the potential to induce frustration. Escape from S− and time-out avoidance offer interesting parallels with fear in the shock escape/avoidance situation and are thus potentially useful to shed light on emotional processes related to frustration in pigeons.

### Persistence

The partial reinforcement extinction effect (PREE) is defined as greater resistance to extinction after acquisition with partial rather than continuous reinforcement. It is a ubiquitous effect that occurs under a wide range of conditions and in many species (Papini, 2014). In birds, the PREE has been found also under a variety of conditions, including massed and spaced training trials. Under massed conditions, training is administered either in a free-operant situation with the key light continuously available for responding (e.g., Nevin, 1988) or with intervals between trials in the order of seconds to a few minutes in discrete-trial situations (Jenkins, 1962; Gonzalez and Champlin, 1974). Massed training conditions can produce behavioral effects that are consistent with emotional memory, but based upon nonemotional mechanisms. When trials are administered in closed temporal proximity, the carry-over sensory or short-term memory traces of rewards consumed in one trial can acquire control over responding during the following trial. Partially reinforced animals can learn to respond in the presence of carry-over events involving both reward and nonreward, whereas continuously reinforced animals only learn to respond in the presence of reward carry-over events. As a result, a shift to extinction is less disruptive to partially reinforced animals than it is to continuously reinforced animals—a matter of stimulus generalization decrement (Hull, 1952).

There is evidence that short intertrial intervals promote the PREE under conditions that do not yield this effect when training is widely spaced. For example, toads (*Rhinella arenarum*, formerly *Bufo*) trained to traverse a runway for water reinforcement exhibit the PREE with 15-s intertrial intervals, but not with 300-s intertrial intervals (Muzio et al., 1992). Couvillon et al. (1980) tested the carry-over hypothesis by interpolating nontarget stimuli between trials with the target stimulus. Pigeons showed single alternation behavior when reinforced and nonreinforced trials alternated, but behavioral patterning was eliminated when the interpolated task was introduced. However, the same interpolated task still yielded a PREE to the target stimulus in pigeons, suggesting that carry-over stimuli may not prevent pigeons from reactivating the memory of previous trial outcomes, a mechanism that is assumed to explain the spaced-trial PREE.

This result is consistent with reports of the spaced-trial PREE in pigeons under both key-pecking (Papini et al., 2002) and runway situations (Roberts et al., 1963; Thomas and Papini, 2003). A similar runway effect was observed in quail with food reward (Buriticá et al., 2013). In these experiments, 50% partial reinforcement training administered at a rate of one trial per day led to greater resistance to extinction than continuous reinforcement. Thomas and Papini (2003) extended these findings in three directions. First, they reported that training with variable reward magnitudes (large and small reward intermixed during acquisition) also yield increased resistance to extinction in widely spaced training in pigeons. Second, they asked whether the pigeon PREE was based on the same mechanisms underlying the PREE in rats, and provided evidence based on drug effects. Three drugs were selected based on published research with rats (see Thomas and Papini, 2003, for references): CDP (a GABAergic drug that eliminates the PREE), haloperidol (a dopamine D2 receptor antagonist that has no significant effect on the PREE), and nicotine (an agonist at acetylcholine receptors that enhances the PREE). Although these drugs affected the spaced-trial runway PREE in pigeons, their effects differed relative to those described in rats. For example, CDP retarded the emergence of the PREE in pigeons, but it did not eliminate it; haloperidol and nicotine, however, did eliminate the PREE in pigeons. Thus, although behaviorally analogous, it is plausible that the PREE is based upon different neurotransmitter systems in rats and pigeons, a possibility that remains to be fully analyzed.

Finally, Thomas and Papini (2003) tested the co-variation of the PREE and MREE within the same experiment, using widely spaced training in the runway situation. Four groups of pigeons received acquisition training with large, partial reinforcement (L/P); large, continuous reinforcement matched for reinforcement (L/Cr); large, continuous reinforcement matched for trials (L/Ct); and small, continuous reinforcement (S/C). L/P acquisition yielded increased resistance to extinction compared to L/Cr and L/Ct acquisition and thus yielded evidence of a PREE. However, S/C extinguished faster than L/Ct, thus revealing a reversed MREE. Such a dissociation between these two effects involving SROs is consistent with the hypothesis that these behavioral phenomena are based upon different mechanisms.

It seems clear that birds show a PREE behaviorally similar to that observed in rats. However, whether the effect is dependent upon the emotional memory of frustrating events remains to be determined. The information available thus far suggests that the mechanisms underlying the PREE in pigeons and rats are different.

### Aggressive Behavior

Evidence consistent with an emotional component stemming from SROs comes from experiments assessing aggressive behavior in birds. Azrin et al. (1966) provided a detailed description of the aggressive responses of pigeons trained in intermittent schedules of food reinforcement, during period of food omission:

Visual observation revealed that attack consisted of strong pecks at the throat and head of the target bird, especially around the eyes. The feathers of the target bird were often pulled out and the skin bruised. The attack was often preceded by a brief period of pacing in front of the wall on which the response key was mounted. Occasionally, the pecking attack was preceded by striking movements of the wing or by a slow swaying approach to the target bird with the head lowered. Frequently, the attack was preceded and accompanied by a deep-throated sound (pp. 194–195).

During appetitive extinction, aggressive responses are directed at another pigeon present in the conditioning box (Azrin et al., 1966; Gentry, 1968; Rilling and Caplan, 1973), at a picture of a pigeon projected on a screen (Yoburn and Cohen, 1979), and at a mirror reflecting the image of the pigeon (Cohen and Looney, 1973). Chickens also behave aggressively when food is present, but made inaccessible (Duncan and Wood-Gush, 1971), and also during appetitive extinction (Kuhne et al., 2011). Azrin et al. (1966) found that the intensity of aggressive responses in pigeons increased with the number of rewards in acquisition and decreased when pigeons were given free access to food. Pigeons also display aggressive behavior in the presence of cues that predict reward omissions (Terrace, 1972). Interestingly, wing flapping, an aggressive response, is prominent during the early stages of visual discrimination training when differential responding has not yet emerged. However, as the pigeon learns the discrimination, wing flapping is reduced and eliminated. This result is consistent with the hypothesis that frustration mediates the relationship between unexpected reward failures and aggressive behavior (Amsel, 1992). Frustration is expected to weaken when the discrimination develops and, as a result, nonreward becomes expected in the presence of the S− and so wing flapping is reduced.

There is also evidence that the vocalizations described by Azrin et al. (1966) in the previous quotation reflect emotional arousal. Rashotte et al. (1975) reported evidence of a vocalization (called “Vocal A” by the authors) that usually accompanied aggressive responses, such as during episodes of wing-striking and pecking at the head region of the target pigeon. These were low-frequency vocalizations in the range between 0 and 2 kHz.

In poultry, it has been observed that preventing the occurrence of natural behaviors may redirect behavior. For example, chickens in animal production settings can peck and remove feathers from other birds to the point of causing bleeding. Feather pecking is usually interpreted as resulting from prevented natural behaviors, including foraging or dust bathing behavior. Careful monitoring of the topography of feather pecks in hens concluded that such pecks resemble those involved in foraging, rather than sand bathing, and thus likely develop out of frustrated opportunities for displaying feeding behaviors (Dixon et al., 2008).

Aggressive responses are routinely induced by periods and signals of nonreinforcement, thus offering a potentially fertile ground to test the notion that birds experience frustration. What is missing in this research is a systematic analysis of the nonreward-induced aggressive behavior in terms of underlying neurobiological factors. Drug and brain manipulations are needed to determine the extent to which these behavioral effects can be attributed to emotional activation.

### Conflict

A number of experiments with pigeons have looked at choice in situation involving different reward magnitudes or probabilities. For example, McDiarmid and Rilling (1965) first reported that pigeons prefer a schedule associated to a less frequent reward to one associated to a more frequent reward if the former involves a shorter delay than the latter. Similarly, pigeons trained to withhold key pecking to earn a large reward, rather than pecking immediately to earn a small reward, failed to inhibit responding and, thus, lost a substantial amount of food (Ainslie, 1974). Such performance can typically be reversed by manipulating either the length of the delay or the magnitude of the reward. Variations of this procedure, known as delay discounting, suggest that pigeons, like other animals, are sensitive to temporal delays such that a large reward is reduced in value if it is presented only after a temporal delay. Although delay discounting is usually interpreted in cognitive terms, it is potentially amenable to an interpretation based on frustration (Amsel, 1992). When there is an immediate reward available in the same session, delayed reward options become less acceptable if they induce frustration.

Research on delay discounting has shown that individual neurons located in the pigeon’s nidopallium caudolaterale, a region considered homologous to the mammalian prefrontal cortex, encode information of both reward delay and magnitude (Kalenscher et al., 2005). Additional unit recordings from the nidopallium caudolaterale indicate that some neurons are maximally active during the interval between the onset of the stimulus and the production of the response. A task that required rapid responding to one stimulus (as fast as possible), but postponing responding to a second stimulus for 1.5 s from stimulus onset, revealed units that increased activity with a rate appropriate to these two types of trials—steeper for the rapid-response stimulus than for the waiting-response stimulus (Kalenscher et al., 2006). Moreover, trials in which the pigeon responded too early or too late, thus missing a reward, were characterized by the unit responding at the incorrect rate. Delay discounting offers an interesting arena to test emotional learning in pigeons.

## Further Comments

These additional comments on avian emotions are organized according to three significant questions suggested by the present review. First, *what is the strength of the evidence for fear and frustration in birds*? The evidence for fear in birds seems stronger than the evidence for frustration. As noted in the introduction, the search and evaluation of evidence for fear in birds starts from a comparison with analogous experiments with mammals, since mammalian fear is perhaps the emotion that is best understood. Like mammals, birds show evidence of similar responses to analogous stimulus conditions. TI, freezing, the acquisition of passive and active avoidance responses, and the effects of benzodiazepine anxiolytics in punished responding all point to substantial similarities with mammalian fear. However, these are just a few pieces in a large puzzle. To have a comprehensive view of the degree of similarity in underlying mechanisms, a starting approach would be to systematically compare two species, under analogous conditions, and in terms of at least four levels of analysis: behavioral, neurobiological (neural circuitry), neurochemical (synaptic transmission), and cell-molecular (synaptic plasticity) (Papini, 2002, 2003, 2008). A case for homology of emotional behavior would be strengthened if the same mechanisms were uncovered at all levels of analysis. By contrast, similarity in behavior supported by different underlying mechanisms would be consistent with independent evolution by homoplasy. Of all the preparations for studying emotional behavior reviewed above, the one that seems most suitable for such a detailed analysis would seem to be punished responding. There are extensive behavioral and neurochemical (psychopharmacological) data in both pigeons and rats that set the basis for a systematic evolutionary approach to a search for avian/mammalian homology in fear.

Although the term “frustration” has been used extensively in the literature, especially in applied research with birds, the evidence for extensive similarities in the adjustment to SROs in birds and mammals is not as strong as it is in the case of fear. In some cases, the behavioral effects are simply different (e.g., SNC and related effects). In other cases, pigeons and rats produce similar behavioral effects, but apparently based upon different mechanisms, as revealed by drug manipulations (PREE). A promising phenomenon that would need to be rescued for analysis with current behavioral and neural techniques is that of extinction-induced aggressive behavior in pigeons (Papini and Dudley, 1997; Papini, 2014).

Another result in need for further analysis is the behavioral dissociation between PREE and MREE in pigeons. For example, a comparison between pigeons and rats in their adjustment to SROs suggests different scaling properties of their behavior. For rats, reward downshift is controlled by the ratio of the reward magnitudes obtained in postshift and those expected from preshift experience, rather than by their absolute magnitude (Papini and Pellegrini, 2006; Pellegrini and Papini, 2007; Pellegrini et al., 2008). For pigeons, however, postshift key-pecking performance is controlled by the absolute magnitude of the preshift reward (Pellegrini et al., 2008). The behavior of pigeons in reward devaluation tasks seems to be under tight control by the long-term memory of the preshift reward magnitudes. Instead, rats exhibit behavioral flexibility in analogous situations. Negative emotions such as anticipatory frustration induced by prior experience with SROs may accelerate the detachment from a signal or location previously paired with reward, a process known as incentive disengagement (Papini, 2003). One function of negative emotions may be to facilitate the disengagement from incentives that are no longer yielding sufficient rewards to support survival and reproductive success.

Second, *what is needed to characterize the relationship between brain and emotion in birds*? A model to imitate is provided by research on spatial learning and hippocampal size and function in birds and mammals (Sherry et al., 1992; Broglio et al., 2015). In this case, there is a fit between structure and function that permits a direct visualization of brain-behavior relationships. This is facilitated by the development of analogous training techniques that can be applied to different species (teleost fish, amphibians, birds, and mammals) and a structure that can be clearly identified in terms of homology across a wide range of species. A similar argument could be made for the relationship between active and passive avoidance learning (in relation to fear), or runway performance under conditions of reward devaluation and extinction (in relation to frustration). Such training techniques have been applied to a variety of vertebrate species using analogous procedures. The key structure in this case would be the amygdala, which, like the hippocampus, has identifiable homologs across vertebrates (Moreno and González, 2007; Vargas et al., 2012). For example, studies with Roman high- and low-avoidance rat strains, selected for active avoidance learning since the 1960s (Driscoll and Bättig, 1982), provide a potential approach to follow. In addition to differences in avoidance learning, these strains also exhibit differences in a variety of emotional behaviors, including other types of avoidance learning, anxiety situations, and also situations involving SROs (Torres and Sabariego, 2014). In general, Roman low-avoidance rats show higher levels of anxiety, SNC, PREE, and the emotional self-medication effect involving increased voluntary consumption of anxiolytics immediately after episodes involving SROs, compared to Roman high-avoidance rats. Gómez et al. (2008) reported that poor one-way avoidance in Roman low-avoidance rats was correlated with low cell density in the basolateral amygdala. Similar selective breeding protocols could be applied to some avian species, including Japanese quail and chickens, for which there are also behavioral techniques to study analogous phenomena (e.g., selective breeding for TI; Jones et al., 1991).

Third, *what information do these studies in birds offer to understand the origin of negative emotions in vertebrates*? The study of fear and frustration in mammals has suggested that the underlying neural mechanisms overlap extensively—the fear = frustration hypothesis (Wagner, 1969; Gray, 1987; Gray and McNaughton, 2000). Comparative research in other vertebrates can help determine whether brain circuits dedicated to these emotions arose simultaneously or sequentially during vertebrate evolution. Based on data on avoidance learning in goldfish (*Carassius auratus*), Papini (2003) suggested that the brain mechanisms underlying frustration could have evolved from fear mechanisms in early mammalian ancestors by a combination of gene duplication and co-option. This is based on several sources of evidence, including the comparative distribution of the SNC effect in vertebrates. SNC has been described in several mammalian species and it has been shown to correlate with behavioral and physiological indicators of emotional activation (Papini et al., 2015). Analogous experiments with teleost fish, amphibians, turtles, and birds have provided no evidence of SNC, with the exception of a single experiment with starlings (Papini, 2014). However, some of the same species that show no evidence of SNC readily provided evidence for avoidance learning: goldfish (Portavella et al., 2003), amphibians (Daneri et al., 2007), and birds (see above). Interestingly, avoidance behavior in goldfish (Portavella et al., 2004) and in toads (Puddington et al., 2016) depends on activity in brain areas homologous to the mammalian amygdala.

Much remains to be done to develop lab techniques to study behaviors clearly related to emotion, following the lead of such studies with mammals. This review suggests several potential candidates for behavioral targets. But, in addition, behavioral effects need to be analyzed at lower mechanistic levels from a comparative perspective to uncover clues on the evolution of vertebrate emotional systems. These goals could be best approached at this time using fear and frustration as model emotions.

## Author Contributions

All the three authors contributed, to varying degrees, in each of the following items: conception of the article, writing of the manuscript, review of form and content, and approval of the final manuscript.

### Conflict of Interest Statement

The authors declare that the research was conducted in the absence of any commercial or financial relationships that could be construed as a potential conflict of interest.
